# NOD2/RICK-Dependent β-Defensin 2 Regulation Is Protective for Nontypeable *Haemophilus influenzae*-Induced Middle Ear Infection

**DOI:** 10.1371/journal.pone.0090933

**Published:** 2014-03-13

**Authors:** Jeong-Im Woo, Sejo Oh, Paul Webster, Yoo Jin Lee, David J. Lim, Sung K. Moon

**Affiliations:** 1 Department of Head and Neck Surgery, David Geffen School of Medicine, University of California Los Angeles, Los Angeles, California, United States of America; 2 Division of Clinical and Translational Research, House Research Institute, Los Angeles, California, United States of America; 3 Center for Electron Microscopy and Microanalysis, University of Southern California, Los Angeles, California, United States of America; University Hospital Schleswig-Holstein, Campus Kiel, Germany

## Abstract

Middle ear infection, otitis media (OM), is clinically important due to the high incidence in children and its impact on the development of language and motor coordination. Previously, we have demonstrated that the human middle ear epithelial cells up-regulate β-defensin 2, a model innate immune molecule, in response to nontypeable *Haemophilus influenzae* (NTHi), the most common OM pathogen, via TLR2 signaling. NTHi does internalize into the epithelial cells, but its intracellular trafficking and host responses to the internalized NTHi are poorly understood. Here we aimed to determine a role of cytoplasmic pathogen recognition receptors in NTHi-induced β-defensin 2 regulation and NTHi clearance from the middle ear. Notably, we observed that the internalized NTHi is able to exist freely in the cytoplasm of the human epithelial cells after rupturing the surrounding membrane. The human middle ear epithelial cells inhibited NTHi-induced β-defensin 2 production by NOD2 silencing but augmented it by NOD2 over-expression. NTHi-induced β-defensin 2 up-regulation was attenuated by cytochalasin D, an inhibitor of actin polymerization and was enhanced by α-hemolysin, a pore-forming toxin. NOD2 silencing was found to block α-hemolysin-mediated enhancement of NTHi-induced β-defensin 2 up-regulation. NOD2 deficiency appeared to reduce inflammatory reactions in response to intratympanic inoculation of NTHi and inhibit NTHi clearance from the middle ear. Taken together, our findings suggest that a cytoplasmic release of internalized NTHi is involved in the pathogenesis of NTHi infections, and NOD2-mediated β-defensin 2 regulation contributes to the protection against NTHi-induced otitis media.

## Introduction

The innate immune system constitutes the body’s first line of defense against pathogens prior to activation of the adaptive immune response [1]. It has become clear that secreted antimicrobial innate immune molecules (AIIMs) such as defensins play an essential role in the innate immune response to pathogens [2]. Defensins are small cationic peptides that selectively form multiple pores to the bacterial membrane, leading to a bactericidal effect [3]. Particularly, β-defensins serve as a first line of defense in the body surface because they are exclusively produced in the keratinocytes and epithelial cells lining the respiratory, gastrointestinal and urinary tracts [2]. In our prior study, we showed that β-defensin 2 (also known as DEFB4 or DEFB4A) is regulated by IL-1 in the human middle ear epithelial cells [4]. We also demonstrated that β-defensin 2 has a potent antimicrobial effect on nontypeable *Haemophilus influenzae* (NTHi), an opportunistic human pathogen [5] and is highly inducible by the NTHi molecules [6], [7].

NTHi is a small Gram-negative bacterium existing as a commensal organism in the human nasopharynx [8] and forming biofilms on human mucosal surfaces [9], [10]. Unlike *H. influenzae* type b, NTHi is nontypeable since it lacks a polysaccharide capsule used for typing and rarely causes life-threatening infections [11]. Nonetheless, NTHi is a clinically important pathogen due to its role in exacerbating chronic obstructive pulmonary disease in adults and causing middle ear infection in children [12], [13]. Middle ear infection, otitis media (OM), is one of the most common pediatric infectious diseases accounting for 25% of antibiotic prescriptions [14]. OM frequently leads to hearing impairment (conductive and sensorineural) in children, impacting their language development during the critical period [15], [16]. Among the major OM pathogens, NTHi is becoming the most common pathogen causing 35–56% of the OM cases due to a pathogenic shift by the recent introduction of pneumococcal vaccination [17], [18].

NTHi is considered as an extracellular pathogen; however, bacterial internalization (or invasion) into the epithelial cells is suggested to contribute to the pathogenesis of NTHi infections because they frequently persist despite antibiotic therapy and recur after asymptomatic periods [19], [20]. NTHi is known to enter host cells through macropinocytosis initiated by cytoskeletal rearrangement [21] and endocytosis mediated by lipid raft or a receptor [22]–[24]. After internalization, some pathogens are able to exist freely in the cytoplasm after inhibition of the phagosomal (endosomal) maturation [25], [26], but intracellular trafficking of NTHi is poorly understood [27].

Among a variety of pathogen recognition receptors (PRRs) sensing pathogen-associated molecules, toll-like receptor 2 (TLR2), a membrane-bound PRR, is known to be required for the recognition of the NTHi molecules [28] even though NTHi is Gram-negative. In addition to a variety of genes involved in inflammation and ion homeostasis [29], [30], the middle ear epithelial cells were shown to up-regulate β-defensin 2 in response to NTHi via TLR2 signaling [7]. NTHi is capable of entering the epithelial cells, but our understanding about the role of cytoplasmic PRR in NTHi-induced β-defensin 2 regulation remains limited. Among the cytoplasmic PRRs, nucleotide-binding oligomerization domain-containing proteins (NODs) are involved in β-defensin 2 regulation [31], [32] and NTHi-induced IL-8 regulation [33]. Thus, we aimed to determine a role of NODs in NTHi-induced β-defensin 2 regulation in the middle ear epithelial cells and its involvement in protection of the middle ear from the OM pathogens.

In this study, we present that internalized NTHi ruptures and escapes from the surrounding membrane into the cytoplasm of the human epithelial cells. Moreover, we show that NOD2 is involved in recognition of the NTHi molecules and up-regulation of β-defensin 2 in the middle ear epithelial cells. Modifications of the epithelial cell membrane appeared to affect NOD2-dependent β-defensin 2 regulation. We also demonstrate that NOD2 deficiency significantly influences middle ear inflammatory reactions and NTHi clearance from the middle ear cavity. This study will enable us to better understand about intracellular trafficking of internalized NTHi and host responses to NTHi in the cytoplasm of the epithelial cells.

## Materials and Methods

### Ethics Statement

All animal experiments in this study were approved by the Institutional Animal Care and Use Committee of the House Research Institute, permitted protocol #HE1131-09-03.

### Reagents

Cytochalasin D (CytD), α-hemolysin, and calcein AM were purchased from Sigma-Aldrich (St. Louis, MO). siRNAs for NOD1 (NM_006092: 5′-GGCCAAAGUCUAUGAAGAUtt-3′), NOD2 (NM_022162: GGCUGAAAUUCAGAAUAUUtt-3′), TLR2 (NM_003264: 5′-GCCUUGACCUGUCCAACAAtt-3′), NLRC4 (NM_021209: 5′-GGUUCAAGCCAAAGUAUAAtt-3′) and RICK (NM_003821: 5′-GGAGAAGAAUUUGCCAAAGtt-3′) were purchased from Life Technologies (Grand Island, NY). Anti-Defb2, anti-NOD2, and anti-β-tubulin antibodies were purchased from Santa Cruz Biotechnology (Dallas, TX).

### Bacterial Culture and Preparation of Bacterial Lysate

NTHi 12 (provided by Dr. Xin-Xing Gu, NIH), originally a clinical isolate from the middle ear fluid of a child with acute OM [34], and GFP-conjugated NTHi 86-028NP (provided by Dr. Lauren Bakaletz, Ohio State University) [35] were used in this study. The bacteria were streaked onto fresh chocolate agar plate and incubated overnight in a 37°C incubator with 5% CO_2_. A single colony was grown in 3 ml of brain heart infusion broth (BHI, Becton Dickinson, Cockeysville, MD), supplemented with hemin (10 µg/ml) and NAD (10 µg/ml). NTHi lysate was prepared as previously described [36]. Briefly, bacteria of a mid-log phase (*A*_600_ = 0.4 to 0.5) were collected after centrifugation at 6,000×g for 30 min, and the bacterial pellet was resuspended in 5 ml of sterile water. After bacterial suspension was sonicated for 2 min in ice-water slurry, the samples were centrifuged at 12,000×g for 10 min at 4°C, and the supernatants were collected. Protein concentrations of the NTHi lysates were determined using a BCA™ protein assay kit (Pierce Biotechnologies, Rockford, IL).

### Animal Experiments and Primary Explant Culture

C57BL/6, TLR2^−/−^ (C.129 (B6)-*Tlr2^tm1Kir^*) and NOD2^−/−^ (B6.129S1-*Nod2^tm1Flv^*) mice were purchased from Jackson Laboratory (Bar Harbor, MA). TLR2^−/−^NOD2^−/−^ mice were generated by intercrossing of TLR2^−/−^ and NOD2^−/−^, and genotypes were determined by PCR primers provided by Jackson Laboratory. All aspects of animal handling were performed according to the approved Institutional Animal Care and Use Committee (IACUC) guidelines. We induced an experimental OM as described [37]. Briefly, animals were anesthetized with intraperitoneal injection of ketamine (80 mg/kg) and xylazine (10 mg/kg). 10^7^ CFU of live NTHi in 5 µl of saline were transtympanically inoculated to the middle ear cavity for histological analysis, and 300 CFU of NTHi, for bacterial clearance analysis. As a control, 5 µl of sterile normal saline was inoculated. Animals were euthanized 24 h after injection, and the temporal bones were dissected and embedded in paraffin for H & E staining and immunolabeling as described [38] (Fig. S1). For bacterial clearance analysis, we collected middle ear effusions, which were cultured in the chocolate agar plates. For primary culture of middle ear epithelial cells, 10 week-old male mice were euthanized and decapitated. The bulla was isolated after sterile dissection, and the bony capsule was carefully removed, preserving the middle ear mucosa. Explants of the middle ear mucosa were plated onto the collagen-coated culture dishes in DMEM (Life Technologies) supplemented with 10% FBS.

### Cell Culture

The human middle ear epithelial cell line (HMEEC) [39] was maintained in a 1∶1 mixture of DMEM and bronchial epithelial basal medium (Lonza, Walkersville, MD) supplemented with bovine pituitary extract (50 µg/ml), hydrocortisone (0.5 µg/ml), hEGF (0.5 ng/ml), epinephrine (0.5 µg/ml), transferrin (10 µg/ml), insulin (5 µg/ml), triiodothyronine (6.5 ng/ml), retinoic acid (0.1 ng/ml), gentamicin (50 µg/ml), and amphotericin-B (50 ng/ml). The Chang cells (the human conjunctival epithelial cell line, CCL-20.2), Hela cells (the human cervical epithelial cell line, CCL-2), and A549 cells (the human lung epithelial cell line, CCL-185) were purchased from the ATCC (Manassas, VA) and maintained in DMEM supplemented with 10% FBS. All cells were cultured at 37°C in a humidified atmosphere with 5% CO_2_.

### Gentamicin Protection Assay and Endocytosis Assay

To determine bacterial internalization, gentamicin protection assays were performed as described [24]. Briefly, suspensions of mid-log phase (*A*_600_ = 0.4 to 0.5) NTHi (2×10^7^ CFU/well) were applied to 50% confluent monolayer of the HMEEC cells and the Chang cells (2×10^5^ cells/well). After 4 h incubation, each well was washed with PBS twice, and 100 µg/ml of gentamicin was added for 2 h to remove the extracellular bacteria. To determine the number of viable intracellular NTHi, all cells were harvested with 0.25% Trypsin-EDTA and were mechanically lysed by vigorous vortexing with small glass pearls for 1 min. After plating the cell lysate on chocolate agar plates, bacterial colonies were counted after 24 h incubation. To determine internalized GFP-conjugated NTHi, fluorescent microscopy (Zeiss Axio Observer Z1 microscope equipped with AxioVision image analyzer (Carl Zeiss, Thornwood, NY)) was performed using cells grown onto a coverslip and fixed with 4% para-formaldehyde solution. For endocytosis assays, 0.5 µM of calcein AM was added to the HMEEC cells after pretreatment of α-hemolysin (0.5∼1.0 µg/ml). After 2 h incubation, cells were washed with PBS and briefly lysed with a NP-40 cell lysis buffer. After the lysates were transferred to a 96-well plate, fluorescence was measured at 490 nm/520 nm using a plate reader.

### Transmission Electron Microscopy

After co-cultured with NTHi for 4 h, cells were fixed for 1 h by immersion in 2.5% glutaraldehyde in 100 mM cacodylate buffer (pH 7.2), scraped, pelleted and processed using a microwave-assisted method as previously described [40]. Epoxy resin-embedded sections were examined and photographed with a CM120 BioTwin transmission electron microscope (FEI, Inc., Hillsboro, OR), operating at 80 kVa. Images were recorded on negative films and then digitized using a flat bed scanner. Images were cropped for publication to emphasize relevant regions in the cell and modifications of the scanned images consisted of adjusting contrast and brightness. All adjustments were applied uniformly to the whole image.

### Quantitative RT-PCR

Quantitative RT-PCR analysis was performed using a FAM™-conjugated probe for β-defensin 2 and a VIC-conjugated probe for cyclophilin with the ABI 7500 Real Time PCR System (Life Technologies) as described [6]. The cycle threshold (CT) values were determined according to the manufacturer’s instructions. CT values were normalized to the internal control (cyclophilin), and the relative quantity of mRNA was determined using the 2^−(ΔΔCT)^ method [41]. Taqman primers and probes for human β-defensin 2 (DEFB4, NM_004942, Hs00175474_m1), TLR2 (NM_003264, Hs00152932_m1), NOD1 (NM_006092, Hs00196075_m1), NOD2 (NM_022162, Hs00223394_m1) and cyclophilin (NM_005729, 4326316E) were purchased from Life Technologies. Primers used for conventional RT-PCR analysis were as follows: 18s rRNA (NR_003286: 5′-GTGGAGCGATTTGTCTGGTT-3′; 5′-CGCTGAGCCAGTCAGTGTAG-3′), mouse Defb2 (NM_010030: 5′-GCCATGAGGACTCTCTGCTC-3′; 5′-GGACAAATGGCTCTGACACA-3′), mouse Defb3 (NM_013756: 5′-GTCATGAGGATCCATTACCTTCT-3′; 5′-CGGGATCTTGGTCTTCTCTATTT-3′), mouse Defb4 (NM_019728: 5′- CTTGCAGCCTTTACCCAAATTAT-3′; 5′-CTAGAACTGGAGTTAGAGAAGGTAATC-3′), NLRC4 (NM_021209: 5′-TGCCCAGAAATCGAAGCCCTGATA-3′; 5′-TTGGAGCAACAAGCCTTCAGCAAG-3′), and RICK (NM_003821: 5′-TCCTGCATGAAATTGCCCTTGGTG-3′; 5′-ATCGTGCTTGATACTGGCCCTTGA-3′).

### ELISA

To measure protein levels of human β-defensin 2 produced by the HMEEC cells, cells were transfected with the NOD2-specific siRNA or the NOD2-expressing construct. Culture supernatants were collected 24 h after exposure to the NTHi lysate. Protein levels of human β-defensin 2 were quantified by ELISA according to the manufacturer’s instructions (Phoenix Pharmaceuticals, Burlingame, CA). Unknown concentrations of the samples were determined by extrapolation to the standard curve of recombinant human β-defensin 2, and data were expressed as pg/ml.

### Plasmid Construction, Luciferase Assay and Gene Silencing

Constructs used for luciferase assays were as follows: pDEFB4/−2625/luc containing the 5′-flanking region (−2625 to +1) of the human β-defensin 2 gene (AF071216) [4], pTAL-luc containing the firefly luciferase gene with a TATA-like promoter region from the Herpes simplex virus thymidine kinase promoter (Clontech, Moutain View, CA), and pNFκB-luc containing multiple copies of the NF-κB consensus sequence fused to pTAL-luc (Clontech). The vector construct expressing human NOD2 was provided by Dr. Neil Warner (University of Michigan). Cells were transfected at ∼60% confluency using the Transit-LT1 Transfection Reagent (Mirus, Madison, WI), and the pRL-TK vector (Promega) was co-transfected for normalization of transfection efficiency as described [42]. Transfected cells were then starved overnight in serum-free DMEM, followed by exposure to the NTHi lysate for 8 h before harvesting. All transfections were carried out in triplicate. After lysing cells, luciferase activity was measured using a luminometer (Pharmingen, La Jolla, CA) after adding luciferase substrates (Promega). For gene silencing, gene-specific siRNAs were transfected using a siPORT NeoFX transfection agent (Ambion) according to the manufacturer’s instruction. As a control, the negative control siRNA (Ambion) was transfected in parallel. siRNA-mediated gene silencing was confirmed by quantitative RT-PCR (Fig. S2).

### Statistics

All experiments were carried out in triplicate and repeated twice. Results were expressed as means ± standard deviation. Statistical analysis was performed using Student’s *t*-test and ANOVA followed by Tukey’s post hoc test using the R2.14.0 software (The R Foundation for Statistical Computing, Vienna, Austria). A value of *p*<0.05 was considered significant.

## Results

### Internalized NTHi Freely Exists in the Cytoplasm of the Human Epithelial Cells

**Figure 1:**
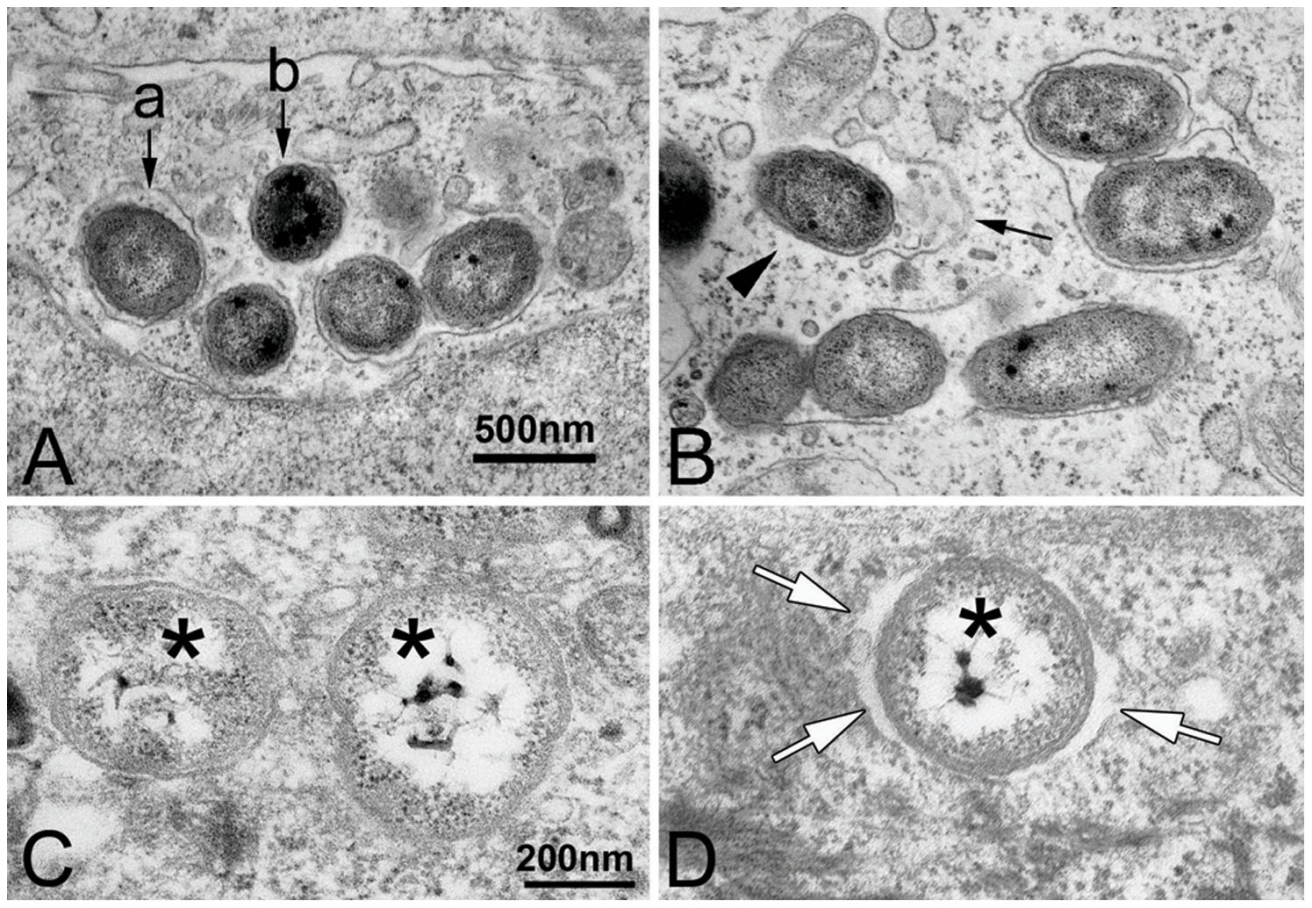
NTHi exists as a free or entrapped form after internalization into the human epithelial cells. (A) Transmission electron microscopic image shows that NTHi is found either surrounded by an enclosing membrane (a) or free in the cytoplasm (b). (B) The membrane (arrow) surrounding the bacterial cell is partially ruptured, and NTHi (arrowhead) appears to be in the process of being released into the cytoplasm of the Chang cells. Scale bar for both images: 500 nm. (C, D) Note that NTHi (asterisks) exists with or without a peribacterial space (white arrows) in the cytoplasm of the HMEEC cells. Scale bar for both images: 200 nm.

NTHi is capable of attaching to and entering a variety of epithelial cells [43], which is initiated by cytoskeletal rearrangement of the host cells [21], [44]. To determine internalization of live NTHi into the human epithelial cells, we performed gentamicin protection assays. When 2×10^5^ of epithelial cells were exposed to 2×10^7^ cfu of live NTHi for 4 h, non-opsonic entry of NTHi appeared at the rate of 4.26±2.33 cfu per 10^3^ of the HMEEC cells and 35.26±15.93 cfu per 10^3^ of the Chang cells. After internalization into the non-professional phagocytes including fibroblasts and epithelial cells, some pathogens such as *Listeria* and *Shigella* are known to escape from the membrane-bound compartment (phagosome or endosome) into the nutrient-rich cytoplasm [25], [26], [45]. To determine intracellular trafficking of internalized NTHi, we performed transmission electron microscopy after exposure of live NTHi to human epithelial cells such as the Chang cells and HMEEC cells. Internalized NTHi was found to exist either surrounded by an enclosing membrane or freely in the cytoplasm of the Chang cells (Fig. 1A). Interestingly, we observed NTHi in the process of being released into the cytoplasm after rupturing the enclosing membrane (Fig. 1B). As shown in Figure 1C and D, NTHi was found to exist with or without a peribacterial space in the cytoplasm of the HMEEC cells. These findings suggest that NTHi is able to compromise maturation of phagosomes and that the cytoplasmic PRRs are required for the recognition of the NTHi molecules in addition to the membrane-bound PRRs such as TLR2 [7], [46].

### A Cytoplasmic Pathogen Recognition Receptor, NOD2 is Involved in Recognition of NTHi

**Figure 2:**
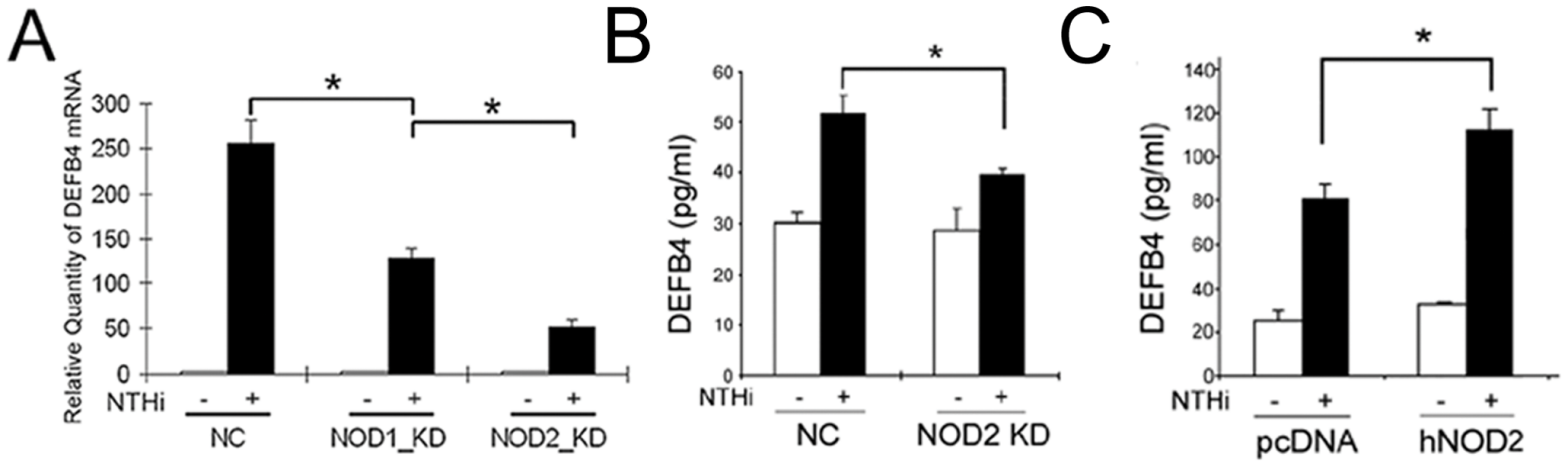
NOD2 contributes to NTHi-induced human β-defensin 2 up-regulation. (A) Quantitative RT-PCR analysis shows that NOD2-specific siRNA inhibits NTHi lysate-induced human β-defensin 2 up-regulation more than NOD1-specific siRNA in the HMEEC cells. ELISA analysis shows that NTHi lysate-induced human β-defensin 2 production is suppressed by the NOD2-specific siRNA (B) but is enhanced by NOD2 overexpression (C) in the HMEEC cells. DEFB4: human β-defensin 2, NC: a control group silenced with a nonspecific negative control siRNA, KD: a group silenced with a gene-specific siRNA, pcDNA: a mock transfection, hNOD2: a construct expressing human NOD2. Results were expressed as fold induction, taking the value of the non-treated group as 1. The experiments were performed in triplicate and repeated twice. Values are given as the mean ± standard deviation (n = 3). *: *p*<0.05.

Among the membrane-bound PRRs, TLR2 plays a major role for host’s recognition of the NTHi molecules [46], [47]. In our prior study, the HMEEC cells were shown to up-regulate human β-defensin 2 in response to the NTHi lysate through the TLR2-dependent signaling pathway [7], but we found that the TLR2-deficient mice preserve the ability to up-regulate mouse Defb2 by intratympanic injection of live NTHi. To determine if a TLR2-independent pathway contributes to NTHi-induced mouse Defb2 up-regulation, the primary middle ear epithelial cells were derived from the TLR2-deficient mice and were exposed to the NTHi lysate. Conventional RT-PCR analysis showed that depletion of TLR2 did not completely block NTHi-induced mouse Defb2 up-regulation (Fig. S3A), suggesting the involvement of the TLR2-independent signaling pathways. As a TLR2-independent pathway, we further investigated cytoplasmic PRRs because NTHi revealed the ability to freely exist in the cytoplasm. Based on the reports showing the involvement of cytoplasmic PRRs such as NOD1 and NOD2 in β-defensin 2 regulation induced by *H. pylori* and muramyl dipeptide [31], [32], we sought to determine if NOD1 and/or NOD2 contribute to NTHi-induced human β-defensin 2 up-regulation in the HMEEC cells. Since NOD1 recognizes peptidoglycan-derived molecules from most Gram-negative bacteria [48] and is required for NTHi-induced IL-8 up-regulation [33], we expected that NOD1 would be mainly responsible for NTHi-induced human β-defensin 2 up-regulation. However, quantitative RT-PCR analysis showed that NOD2-specific siRNA inhibits NTHi-induced human β-defensin 2 up-regulation (∼80%) more than NOD1-specific siRNA (∼50%) (Fig. 2A), indicating that NOD2 is more significantly involved in NTHi-induced human β-defensin 2 up-regulation in the HMEEC cells.

To further determine the requirement of NOD2 for NTHi-induced human β-defensin 2 up-regulation, we performed ELISAs for quantitation of human β-defensin 2 protein levels after silencing or overexpressing of NOD2 in the HMEEC cells. As shown in Figure 2B and 2C, siRNA-mediated inhibition of NOD2 expression appeared to suppress NTHi-induced human β-defensin 2 production (∼55%) while overexpression of NOD2 was found to augment human β-defensin 2 production (∼45%). These findings indicate the critical involvement of NOD2 in the human β-defensin 2 production in response to the NTHi lysate, which plays an important role for the antimicrobial defense in the HMEEC cells. Next, we sought to determine how silencing of both TLR2 and NOD2 affects NTHi-induced human β-defensin 2 up-regulation. Cells were transfected with either or both TLR2-specific and NOD2-specific siRNAs and were exposed to the NTHi lysate. Quantitative RT-PCR analysis showed that NTHi-induced human β-defensin 2 up-regulation was completely inhibited when both TLR2 and NOD2 were simultaneously silenced (Fig. S3B), indicating that NOD2 signaling serves as a TLR2-independent pathway for NTHi-induced human β-defensin 2 up-regulation.

### NOD2-associated Molecules Contribute to NTHi-induced β-defensin 2 Up-regulation

**Figure 3:**
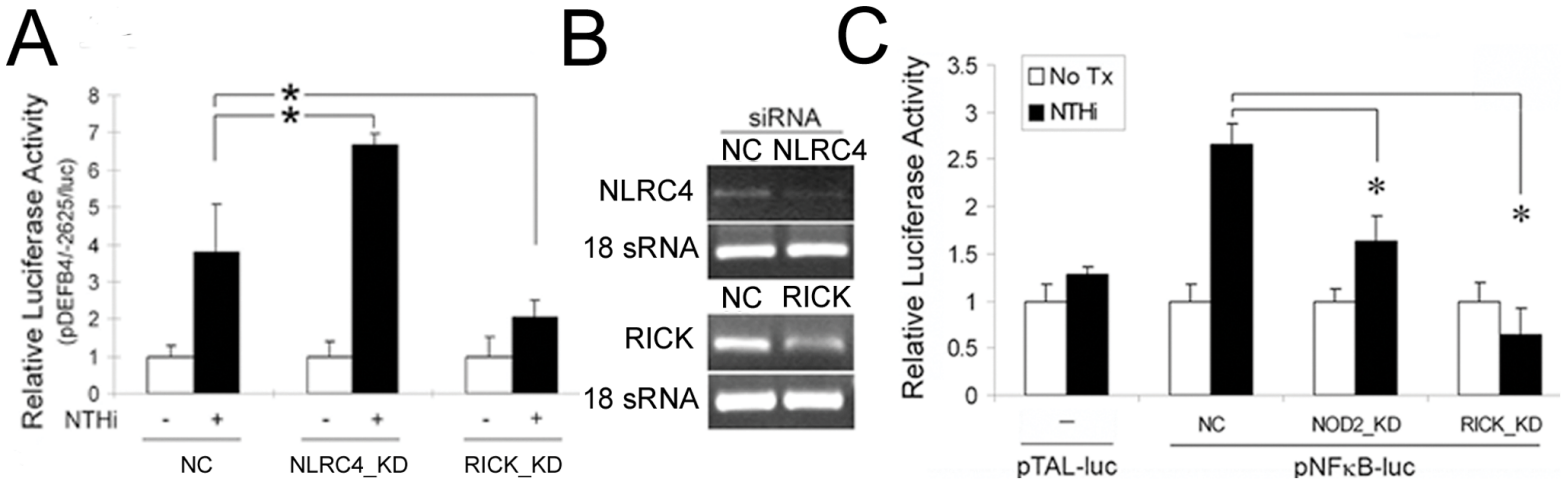
Involvement of NLRC4 and RICK in NTHi-induced human β-defensin 2 up-regulation. (A) Luciferase assays show that NTHi lysate-induced human β-defensin 2 up-regulation is enhanced by silencing of NLRC4 (a NOD2 inhibitor) but is inhibited by silencing of RICK that is downstream to NOD2 in the HMEEC cells. NC: a control group transfected with a nonspecific siRNA, KD: a group transfected with a gene-specific siRNA. (B) RT-PCR analysis showing siRNA-mediated inhibition of NLRC4 and RICK expression in the HMEEC cells. (C) Luciferase assays demonstrate that NTHi lysate-induced NF-κB activation is inhibited by the siRNAs specific to NOD2 and RICK in the HMEEC cells. pTAL-luc: a control vector containing the firefly luciferase gene with a TATA-like promoter region from the Herpes simplex virus thymidine kinase promoter, pNFκB-luc: a vector containing multiple copies of the NF-κB consensus sequence fused to pTAL-luc, Tx: treatment. Results were expressed as fold-induction, taking the value of the non-treated group as 1. The experiments were performed in triplicate and repeated twice. Values are given as the mean ± standard deviation (n = 3). *: *p*<0.05.

To determine the signaling pathway of NOD2 for NTHi-induced human β-defensin 2 up-regulation, we sought to explore the involvement of NOD2-associated signaling molecules such as NLRC4 and RICK. It has been known that the NLRC4 (NLR Family CARD Domain-Containing Protein 4), also known as CARD12 (caspase recruitment domain-containing protein 12), inhibits NOD2 activation through a NOD-NOD interaction [49]. RICK (RIPK2) is a CARD-containing serine/threonine kinase, associated with NOD2 through CARD-CARD interactions [50]. Activation of RICK by virtue of binding to an activated NOD2 receptor leads to the activation of downstream IκK complex through polyubiquitylation of IκKγ. To determine if NLRC4 affects NTHi-induced human β-defensin 2 up-regulation, NLRC4-specific siRNA was transfected to the HMEEC cells with a luciferase-expressing vector containing a 5′-flanking region of DEFB4 (pDEFB4/−2625/luc) as described previously [4]. As shown in Figure 3A, luciferase assays showed that silencing of NLRC4 enhances NTHi-induced human β-defensin 2 up-regulation, indicating that NOD2-NLRC4 interaction interferes with NOD2-mediated human β-defensin 2 up-regulation in response to the NTHi lysate. In contrast, silencing of RICK was found to inhibit NTHi-induced human β-defensin 2 up-regulation. Next, we sought to determine if NOD2/RICK signaling affects NTHi-induced NF-κB activation because NF-κB activation is required for human β-defensin 2 regulation [51]. After silencing of either NOD2 or RICK, we performed luciferase assays using pNFκB-luc, a reporter construct of NF-κB activation. As shown in Figure 3C, inhibition of NOD2 and RICK expression appeared to significantly suppress NTHi-induced NF-κB activation. Altogether, these findings suggest that NOD2/RICK-dependent NF-κB activation significantly contributes to human β-defensin 2 up-regulation in response to the NTHi molecules in the cytoplasm of the HMEEC cells.

### Membrane Modification Affects NTHi-induced β-defensin 2 Up-regulation

**Figure 4:**
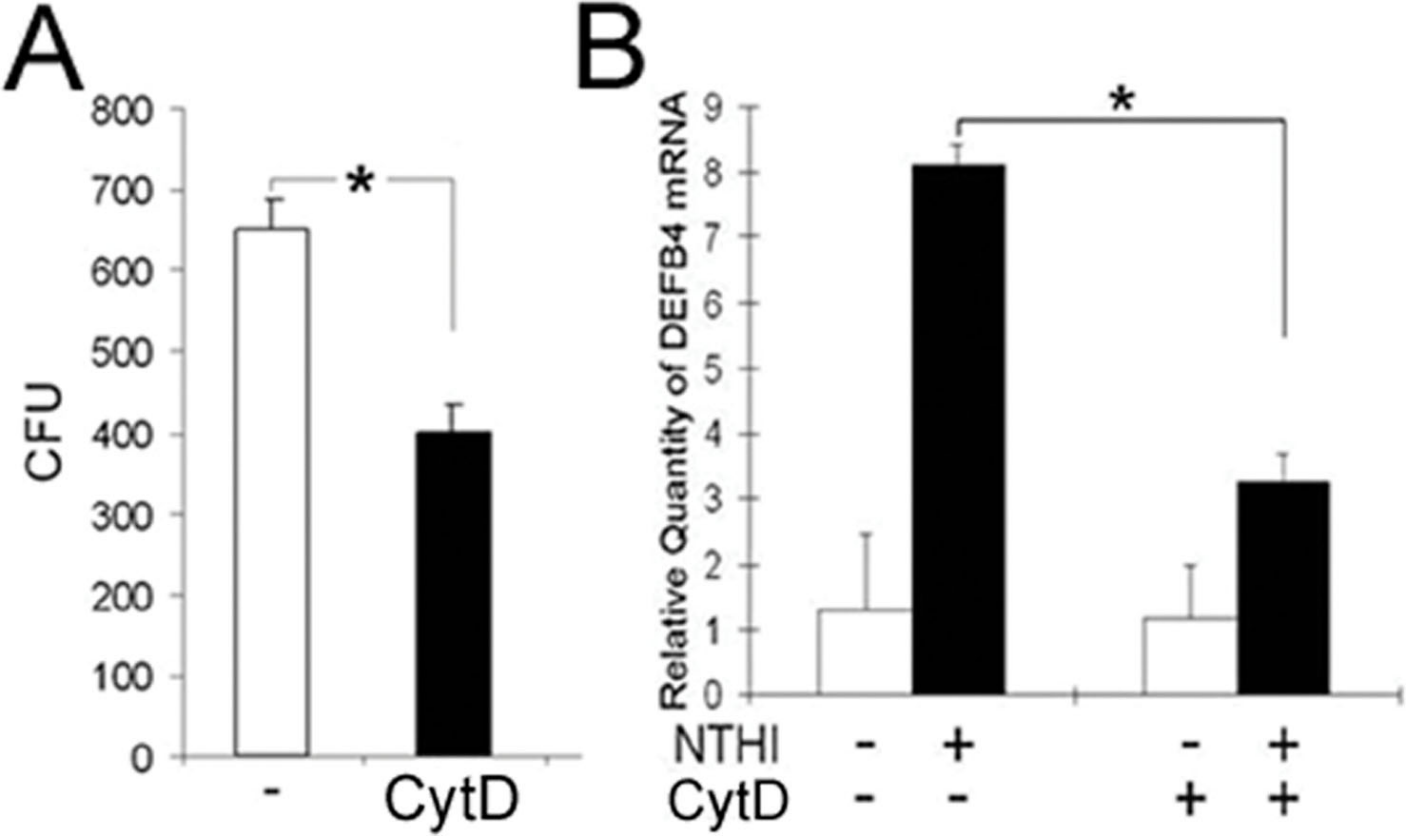
Actin polymerization is involved in NTHi-induced human β-defensin 2 up-regulation. (A) Gentamicin protection assays show that cytochalasin D (CytD), an inhibitor of actin polymerization, inhibits internalization of live NTHi into the HMEEC cells. (B) Quantitative RT-PCR analysis shows that NTHi lysate-induced human β-defensin 2 up-regulation is suppressed by the treatment with CytD in the HMEEC cells. DEFB4: human β-defensin 2. Results were expressed as fold-induction, taking the value of the non-treated group as 1. The experiments were performed in triplicate and repeated twice. Values are given as the mean ± standard deviation (n = 3). *: *p*<0.05.

Since cytoskeletal rearrangement is known to be required for NTHi entry to human airway epithelial cells [21], we sought to determine the involvement of actin polymerization in NTHi internalization into the HMEEC cells. We performed gentamicin protection assays after pretreatment with cytochalasin D (CytD), an inhibitor of actin polymerization. As expected, the HMEEC cells appeared to suppress NTHi internalization (∼35% reduction) upon exposure to CytD (Fig. 4A). Next, we performed quantitative RT-PCR analysis after the HMEEC cells were exposed to the lysate of NTHi to determine if inhibition of actin polymerization affects NTHi-induced human β-defensin 2 up-regulation. As shown in Figure 4B, CytD appeared to inhibit NTHi-induced human β-defensin 2 up-regulation, indicating the involvement of actin polymerization in NTHi-induced human β-defensin 2 up-regulation in the HMEEC cells.

**Figure 5:**
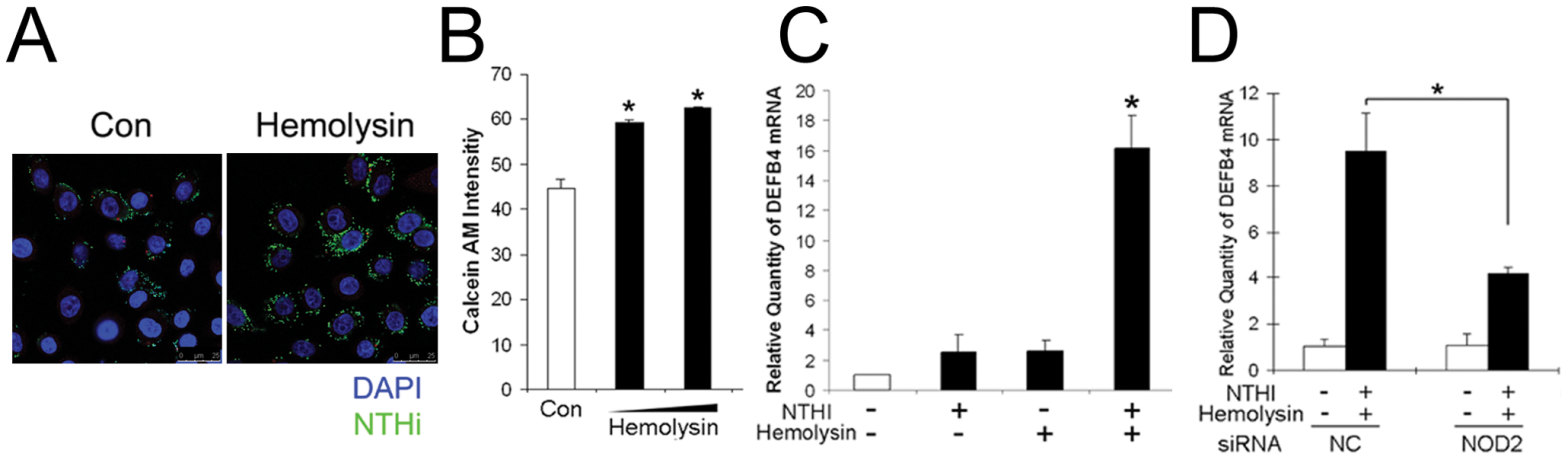
Membrane pore formation enhances NOD2-dependent human β-defensin 2 up-regulation. (A) Fluorescent microscopic images showing that α-hemolysin, a pore-forming toxin, enhances internalization of live NTHi (green) in the HMEEC cells. (B) An influx of calcein AM into the HMEEC cells was increased by α-hemolysin. (C) Quantitative RT-PCR analysis shows that α-hemolysin markedly enhances human β-defensin 2 up-regulation induced by a suboptimal dose (1 µg/ml) of NTHi lysate in the HMEEC cells. DEFB4: human β-defensin 2. (D) Note that silencing of NOD2 inhibits α-hemolysin-mediated enhancement of NTHi lysate-induced human β-defensin 2 up-regulation in the HMEEC cells. NC: a control group transfected with a nonspecific siRNA, KD: a group transfected with a gene-specific siRNA. Results were expressed as fold-induction, taking the value of the non-treated group as 1. The experiments were performed in triplicate and repeated twice. Values are given as the mean ± standard deviation (n = 3). *: *p*<0.05.

Bacterial pore-forming toxins are important virulent factors, but there is controversy regarding to their effects on bacterial internalization. For example, listeriolysin O mediates internalization of *L*. *monocytogenes* into human hepatocytes [52] while α-hemolysin interferes with integrin-mediated internalization *S. aureus* [53]. To determine if pore formation affects NTHi internalization into the HMEEC cells, we performed gentamicin protection assays with GFP-conjugated NTHi after pretreatment of the cells with α-hemolysin, a cholesterol-binding toxin. The HMEEC cells were exposed to 500 ng/ml of α-hemolysin for 3 h, which appeared to insignificantly affect cell viability (Fig. S4D, E). Notably, α-hemolysin appeared to markedly augment NTHi internalization into the HMEEC cells (Fig. 5A). α-Hemolysin-mediated augmentation of NTHi internalization seemed to be associated with actin polymerization (data not shown), but further studies are needed to decipher the molecular mechanism involved. Pneumolysin-induced pore formation is known to increase an uptake of the NTHi molecules, resulting in enhancement of NOD1-mediated signaling [33]. Consistently, endocytosis assays showed that an intracellular influx of calcein AM increases ∼30% by α-hemolysin treatment (Fig. 5B). To further determine if α-hemolysin affects NTHi-induced human β-defensin 2 up-regulation, we performed quantitative RT-PCR analysis after pretreatment with α-hemolysin. A suboptimal dose (1 µg/ml) of the NTHi lysate insignificantly affected human β-defensin 2 regulation, but α-hemolysin appeared to remarkably potentiate human β-defensin 2 up-regulation induced by a suboptimal dose of the NTHi lysate (Fig. 5C). Furthermore, silencing of NOD2 expression was found to inhibit α-hemolysin-mediated enhancement of NTHi-induced human β-defensin 2 up-regulation (Fig. 5D), suggesting pore formation enhances NOD2-mediated human β-defensin 2 up-regulation in response to NTHi molecules. Altogether, these findings indicate that membrane modification affects NOD2-mediated human β-defensin 2 up-regulation through influencing NTHi entry into the epithelial cells.

### NOD2 Deficiency Delays NTHi Clearance from the Middle Ear Cavity

**Figure 6:**
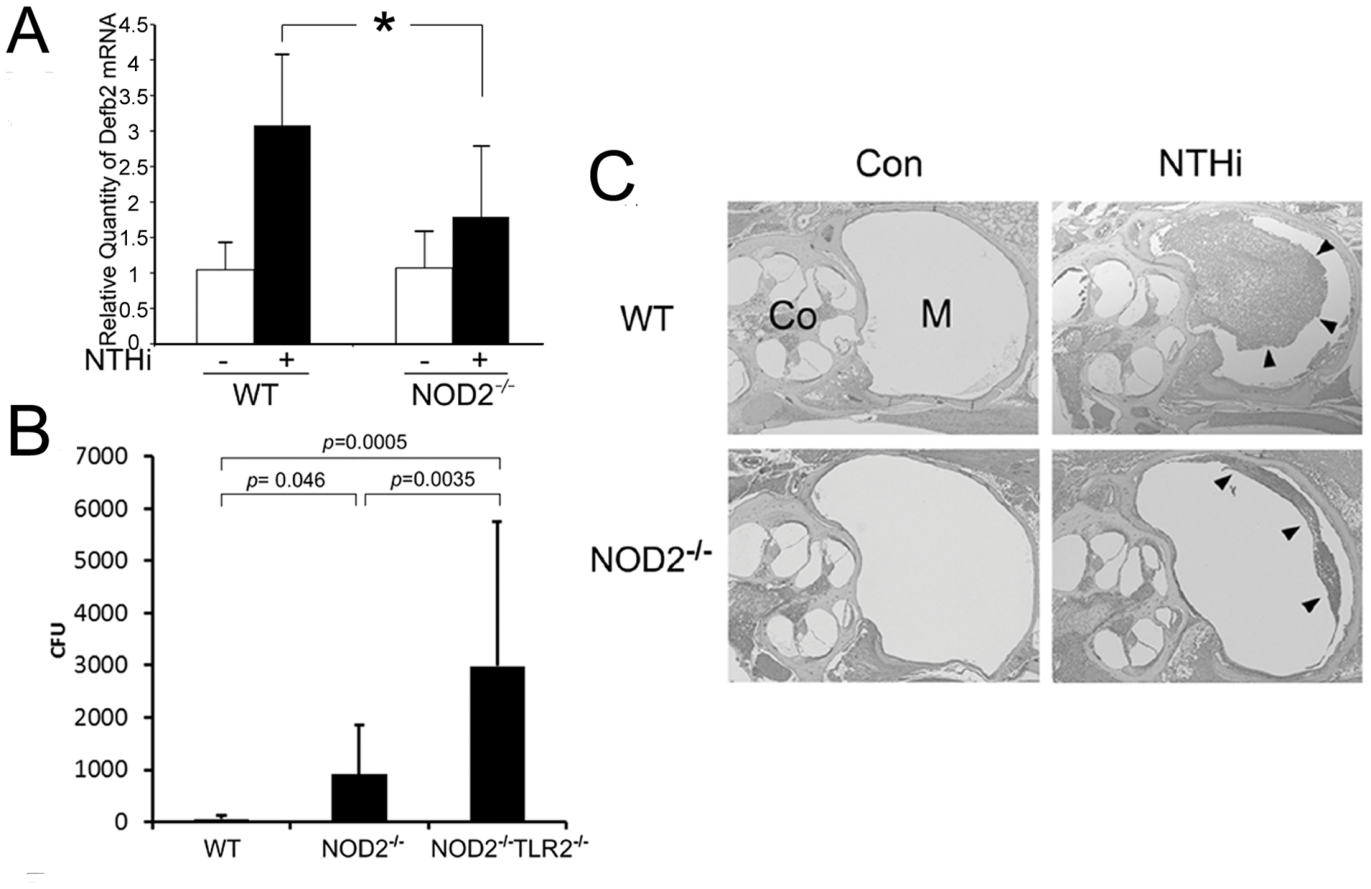
NOD2 is involved in bacterial clearance from the mouse middle ear. (A) Quantitative RT-PCR analysis shows that NOD2-deficient middle ear epithelial cells less up-regulate mouse Defb2 in response to the NTHi molecules, compared to the wild type cells. Values are given as the mean ± standard deviation (n = 3). *: *p*<0.05. (B) Bacterial clearance analysis shows that the number of survived NTHi after intratympanic injection is higher in the NOD2-deficient mice compared to the wild type mice. It is noted that intratympanic NTHi replicates 10-fold in the NOD2/TLR2-deficient mice (NOD2^−/−^TL2^−/−^). Values are given as the mean ± standard deviation (n = 20). (C) Histological analysis shows that middle ear inflammation in response to intratympanic injected NTHi is less severe in the NOD2-deficient mice (NOD2^−/−^) than that in the wild type mice (WT). Arrowheads: middle ear effusion with infiltration of inflammatory cells, M: middle ear cavity, Co: cochlea. Original magnification: x50.

Since we demonstrated a potent antimicrobial effect of human β-defensin 2 on NTHi [5], we sought to determine if NOD2 deficiency influences the pathogenesis of middle ear infection. In addition to our prior study showing that lysozyme deficiency inhibits pneumococcal clearance from the middle ear cavity [37], MyD88 deficiency is known to delay resolution of NTHi-induced otitis media [54]. However, it is poorly understood how NOD2 deficiency affects middle ear inflammation and NTHi clearance from the middle ear cavity. The primary mouse middle ear epithelial cells derived from NOD2-deficient mice were exposed to the NTHi lysate after overnight starvation. As shown in Figure 6A, NOD2-deficient middle ear epithelial cells appeared to inhibit mouse Defb2 up-regulation in response to the NTHi lysate, compared to the wild type control. Next, we sought to determine if NOD2 affects NTHi clearance from the middle ear after intratympanic injection of live NTHi. The lavage of the mouse middle ear cavity was collected 24 h after injection and was cultured to count the number of live NTHi. As shown in Figure 6B, live NTHi was hardly found in the middle ear cavity of the wild type mice 24 h after intratympanic injection. In contrast, intratympanic NTHi appeared to replicate 3-fold in the NOD2-deficient mice and 10-fold in the NOD2/TLR2-deficient mice. Histological analysis of the temporal bones was performed after live NTHi was intratympanically injected into the mouse middle ear cavity. Interestingly, H & E staining showed that middle ear effusion and infiltration of inflammatory cells were less severe in the NOD2-deficient mice than the wild type mice (Fig. 6C), suggesting the involvement of NOD2 in recruitment of inflammatory cells, but further studies are necessary to reveal its molecular mechanism. Taken together, these findings suggest that NOD2 is critically involved in β-defensin 2 production in recognition of cytoplasmic NTHi molecules, which may significantly contribute to the antimicrobial defense of the middle ear as a TLR2-independent pathway.

## Discussion

In this study, we, for the first time, demonstrated the ability of internalized NTHi to escape from the phagosome into the cytoplasm of the human epithelial cells. We also showed that NOD2, a cytoplasmic PRR, is critically involved in recognition of the NTHi molecules, resulting in human β-defensin 2 up-regulation in the HMEEC cells. Membrane modifications such as actin polymerization and α-hemolysin-induced pore formation were shown to influence NOD2-mediated human β-defensin 2 regulation through affecting NTHi entry. Moreover, in a mouse model, we found that NOD2 deficiency attenuates middle ear inflammatory reactions induced by intratympanic injection of live NTHi and delays NTHi clearance from the middle ear cavity, implying an essential role of NOD2 in antimicrobial defense of the middle ear epithelium.

After internalization to the mammalian cells, pathogens are known to usually reside in a membrane-bound compartment, a phagosome (an endosome or a vacuole) [55]. Pathogens inside the phagosome are subject to degradation in the process of phagosomal maturation including acidification and proteolysis by fusion with lysosomes [56], [57]. However, some pathogens are able to survive in the intracellular environment through blocking phagosomal maturation [58], [59] or escaping into the cytoplasm [45]. Internalized NTHi was found within a membrane-bound compartment, which is positive for endosomal antigens [60], but is not colocalized with an autophagy marker, LC3 [22]. Our electron microscopic images showed that internalized NTHi is able to exist either freely in the cytoplasm or surrounded by the membrane in the human epithelial cells. Surprisingly, we observed NTHi in the process of being released into the cytoplasm after rupturing the enclosing membrane. Phagosomal membranes are disrupted by pore-forming toxins [61] and rapid delivery of low pH [40], but further understanding how internalized NTHi compromises phagosomal maturation awaits more studies.

Like TLRs, the leucine-rich repeat domains of NOD1 and NOD2 enable the host to recognize bacterial molecules. NOD1, also known as caspase recruitment domain-containing protein 4 (CARD4), recognizes the peptidoglycan-derived γ-D-glutamyl-meso-diaminopimelic acid from Gram-negative bacteria [48]. In contrast, NOD2, also known as CARD15, recognizes peptidoglycan-derived muramyl dipeptide (MDP) from both Gram-positive and Gram-negative bacteria [62]. It is poorly understood how these cytoplasmic PRRs are able to detect NTHi molecules from the extracellular milieu. Cytoskeletal rearrangement is critically involved in NTHi internalization into the respiratory epithelial cells [21], [44]. NTHi is also known to release outer membrane vesicles during co-culture with host tissues, which directly interact with host cell membrane [57]. Moreover, type IV secretion system in *H. pylori* [63] and the membrane pore formation by a cholesterol-binding toxin in *S. pneumoniae* [33] are known to be involved in internalization of bacterial molecules. Consistently, our result showed that membrane modifications affect NTHi internalization, leading to an influence on NOD2-mediated human β-defensin 2 regulation.

NOD1 is known to be involved in *H*. *influenzae*-induced IL-8 up-regulation in the respiratory epithelial cells [33]. Although NOD1 is the receptor that recognizes peptidoglycan-derived molecules from most Gram-negative bacteria, we found that NOD2 is more importantly involved in the recognition of NTHi molecules than NOD1. We suggest that these findings agree with the requirement of TLR2, not TLR4, for the recognition of NTHi even though NTHi is Gram-negative [46], [47]. NTHi is unique because it contains only atypical lipooligosaccharide, not LPS (the common cell wall component of most Gram-negative enterobacteria). The major ligand for NOD2 is MDP; however, considering that NOD2 is able to interact with non-MDP ligands such as MurNAc-L-Ala-γ-D-Glu-L-Lys and sulfogalactoceramide [64], [65], the NTHi-derived NOD2-specific ligand remains to be identified.

In this study, we have used live NTHi for the experiments of bacterial internalization and bacterial clearance from the middle ear cavity because only live NTHi is able to invade host cells and induce middle ear infections. However, the NTHi lysate has been used for in vitro stimulation of the epithelial cells in this study because (1) live NTHi was found to up-regulate human β-defensin 2 with high variations (Fig. S4B); and (2) the NTHi lysate appeared to more significantly up-regulate human β-defensin 2 expression than the same CFUs of live NTHi as shown in Fig. S4C. Although bacterial lysates are not a perfect model, the NTHi lysate is commonly used for the study of host-pathogen interactions as shown in our prior studies [7], [36], [38], [42] and other studies [46], [66], [67] because live bacteria inhibit viability of mammalian cells. Furthermore, it is suggested that the NTHi lysate is able to up-regulate human β-defensin 2 expression more stably and significantly than live NTHi since a variety of bacterial molecules inside bacteria are able to directly access cytoplasmic PRRs after bacterial lysis. [68].

Previously, we have demonstrated that AIIMs have a potent bactericidal effect on the common OM pathogens such as NTHi [5]. Among a diversity of the AIIMs, β-defensins are produced by the mammalian epithelial cells lining the body surface [69]. Particularly, we selected β-defensin 2 as a model antimicrobial peptide because it effectively kills Gram-negative pathogens and is highly inducible in response to challenging pathogens [2]. Multiple signaling pathways are known to orchestrate the regulation of β-defensin 2, including toll/IL-1 receptor (TIR)-dependent NF-κB activation [70], IL-17-dependent JAK signaling [71] and protease-activated receptor 2 (PAR2)-dependent NF-κB activation [72]. In our prior studies, we showed that the human middle ear epithelial cells up-regulate human β-defensin 2 in response to IL-1 and NTHi lysate via Src/ERK signaling [4] and TLR2/MyD88 signaling [7], in addition to the synergistic effect of IL-1 and NTHi lysate [6]. NOD1 and NOD2 are also known to be involved in β-defensin 2 regulation induced by *H. pylori* and MDP, respectively [31], [32]. In this study, we found that NOD2 signaling importantly contributes to NTHi-induced human β-defensin 2 regulation as a TLR2-independent pathway. Although NOD2, in the absence of MDP, is known to negatively regulate TLR4 signaling [73], NOD2 and TLR2 signaling are suggested to be non-redundant to each other for the NTHi recognition because NODs signaling is rather enhanced by the up-regulation of RICK when TLRs signaling is refractory to TLR agonists [74]. Moreover, NOD2 signaling is tightly regulated to avoid over-stimulation. For example, peptidoglycan-activated NLRC4 interferes with the activation of NOD2, functioning as a negative feedback [49]. Consistently, our results showed that inhibition of NLRC4 enhances NTHi-induced human β-defensin 2 up-regulation, suggesting that NLRC4 serves as a negative regulator for human β-defensin 2 production because high levels of human β-defensin 2 are rather cytotoxic to host cells [75].

In addition to susceptibility to Crohn’s disease and Blau syndrome [76], NOD2 polymorphisms are associated with susceptibility to mycobacterial infections [77], [78]. NOD2-deficient mice are also known to be susceptible to *E. coli*-induced lung infection [79] and exhibit impaired intestinal clearance of Citrobacter [80]. In contrast, NOD2 deficiency insignificantly affected immune reactions to *Y*. *enterocolitica* [81] and paradoxically made mice resistant to *Ehrlichia* infection due to an increase in IL-10 expression and a decrease in CD8+ T cells [82]. Since NTHi is not frequently internalized into the middle ear epithelial cells *in vitro*, we expected that NOD2 deficiency rarely influences bacterial clearance from the middle ear. On the contrary to our expectation, NOD2 deficiency was revealed to significantly impact middle ear inflammatory reactions and NTHi clearance from the middle ear, which agrees with the reports that NTHi internalization is associated with intractable acute OM [83] and that NOD2 expression is up-regulated in the middle ear mucosa of the patients with chronic OM [84]. Our findings indicate that NTHi internalization and NOD2 signaling are significantly involved in the pathogenesis of experimental OM, but further studies are needed to determine an association of NOD2 polymorphisms with recurrence and chronicity of NTHi infections.

Recently, HUGO Gene Nomenclature Committee (HGNC) has changed the official gene name of human β-defensin 2 from DEFB4 to DEFB4A. Moreover, there exists controversy in a murine ortholog of DEFB4A (www.genecards.org/cgi-bin/carddisp.pl?gene=DEFB4A&ortholog=all#orthologs; www.informatics.jax.org/marker/MGI:1338754). In this study, the Defb2 gene has been used as a mouse model of the human β-defensin 2 gene because it is inducible in response to pro-inflammatory stimuli [85], but Defb2 may not reflect the ortholog of DEFB4A. Defb3 [86] and Defb4 [87] seem to be closer to the murine ortholog of DEFB4 since they share more than 40% identical peptides with DEFB4A; however, the real murine ortholog of DEFB4 still remains controversial. As expected, Defb4 was up-regulated in response to the NTHi lysate in the middle ear epithelial cells derived from the wild type mice (Fig. S4A). Moreover, NOD2 deficiency appeared to inhibit NTHi-induced up-regulation of Defb4, which may contribute to delayed bacterial clearance from the middle ear in NOD2-deficient mice. Unexpectedly, Defb3 was insignificantly expressed in the murine middle ear epithelial cells (data not shown), but further studies are necessary to determine a role of the DEFB4 ortholog in experimental OM.

In conclusion, the present study has revealed the ability of NTHi to escape from the phagosome to the epithelial cytoplasm, which may importantly contribute to the pathogenesis of NTHi-induced OM. To defend intracellular NTHi, the middle ear epithelial cells are suggested to be capable of recognizing the NTHi molecules inside the cytoplasm via a NOD2/RICK-dependent signaling pathway, resulting in the induction of antimicrobial molecules. We are only at the beginning of our understanding of the molecular mechanism involved in NTHi internalization and host response, but this study will provide us with a scientific basis for a new therapeutic strategy to manage NTHi infectious diseases.

## Supporting Information

Figure S1 **NTHi induces β-Defensin 2 up-regulation in the middle ear epithelial cells.** (A) An inferior view of the mouse skull base shows that the anatomic relationship of the bulla and Eustachian tube. HP: hard palate, UT: upper teeth, Bu: bulla, FM: foramen magnum, An: anterior, R: right. (B) Schematic illustration of tubotympanum. PO: pharyngeal orifice of Eustachian tube, TO: tympanic orifice of Eustachian tube, TM: tympanic membrane, EAC: external auditory canal, #: the mucosal area nears the tubal orifice for immunolabeling. (C) Immunolabeling shows increased production of mouse Defb2 upon exposure to live NTHi with infiltration of inflammatory cells and mucosal edema. Con: a saline-treated control, ME: middle ear mucosa, TB: temporal bone. Original magnification: x200. (D) Quantitative RT-PCR analysis shows that the NTHi lysate up-regulates human β-defensin 2 in a time dependent manner in the HMEEC cells. E) Luciferase assays show that the NTHi lysate up-regulates human β-defensin 2 expression in the human epithelial cell lines such as the HMEEC cells, the A549 cells and the HeLa cells. DEFB4: human β-defensin 2, Tx: treatment. Results were expressed as fold-induction, taking the value of the non-treated group as 1. Values are given as the mean ± standard deviation (n = 3). *: *p*<0.05.
(DOCX)

Figure S2 **siRNA-mediated silencing of NOD1, NOD2 and TLR2.** Quantitative RT-PCR analysis (A, B) and immunoprecipitation (C) are showing siRNA-mediated silencing of NOD1, NOD2 and TLR2 in the HMEEC cells. NC: a control group silenced with a nonspecific negative control siRNA, KD: a group silenced with a gene-specific siRNA, Double KD: simultaneous silencing of NOD2 and TLR2, IP: immunoprecipitation, WB: western blotting.
(DOCX)

Figure S3 **TLR2 and NOD2 are required for NTHi-induced human β-defensin 2 up-regulation.** (A) RT-PCR analysis shows that TLR2 deficiency (TLR2^−/−^) does not completely block NTHi lysate-induced mouse Defb2 up-regulation in the mouse middle ear epithelial cells. WT: wild type, 18s: 18s rRNA. (B) NTHi lysate-induced human β-defensin 2 up-regulation is blocked when both TLR2 and NOD2 are simultaneously silenced in the HMEEC cells. NC: a control group silenced with a nonspecific negative control siRNA, KD: a group silenced with a gene-specific siRNA.
(DOCX)

Figure S4 (A) RT-PCR analysis shows that mouse Defb4 is up-regulated in response to the NTHi lysate in the primary middle ear epithelial cells derived from wild type mice (WT). In contrast, it is noted that NOD2 deficiency (NOD2^−/−^) inhibits NTHi-induced up-regulation of Defb4. 18s: 18s rRNA. (B) Quantitative RT-PCR analysis shows that the HMEEC cells up-regulate human β-defensin 2 expression in response to live NTHi in a dose-dependent manner, which fails to show a statistical significance due to a high variance. (C) Note that human β-defensin 2 is up-regulated by the lysate (5.75±1.25 µg/ml) extracted from 5×10^7^ CFU of NTHi more significantly than the same CFUs of live NTHi. *: *p*<0.05. MTT assays (D) and MultiTox-Glo™ Multiplex Cytotoxicity Assays (Promega) (E) show that the viability of the HMEEC cells is not affected by exposure of 500 ng/ml of α-hemolysin for 5 h. *: *p*<0.05.
(DOCX)
