# Supplementary material for: NOD2/RICK-Dependent β-Defensin 2 Regulation Is Protective for Nontypeable Haemophilus influenzae-Induced Middle Ear Infection
Source: PLoS One. 2014 Mar 13;9(3):e90933. doi: 10.1371/journal.pone.0090933 (PMC3953203; doi:10.1371/journal.pone.0090933)
Supplement: Figure S1 — NTHi induces β-Defensin 2 up-regulation in the middle ear epithelial cells. (A) An inferior view of the mouse skull base shows that the anatomic relationship of the bulla and Eustachian tube. HP: hard palate, UT: upper teeth, Bu: bulla, FM: foramen magnum, An: anterior, R: right. (B) Schematic illustration of tubotympanum. PO: pharyngeal orifice of Eustachian tube, TO: tympanic orifice of Eustachian tube, TM: tympanic membrane, EAC: external auditory canal, #: the mucosal area nears the tubal orifice for immunolabeling. (C) Immunolabeling shows increased production of mouse Defb2 upon exposure to live NTHi with infiltration of inflammatory cells and mucosal edema. Con: a saline-treated control, ME: middle ear mucosa, TB: temporal bone. Original magnification: x200. (D) Quantitative RT-PCR analysis shows that the NTHi lysate up-regulates human β-defensin 2 in a time dependent manner in the HMEEC cells. E) Luciferase assays show that the NTHi lysate up-regulates human β-defensin 2 expression in the human epithelial cell lines such as the HMEEC cells, the A549 cells and the HeLa cells. DEFB4: human β-defensin 2, Tx: treatment. Results were expressed as fold-induction, taking the value of the non-treated group as 1. Values are given as the mean ± standard deviation (n = 3). *: p<0.05. (DOCX) [file pone.0090933.s001.docx]

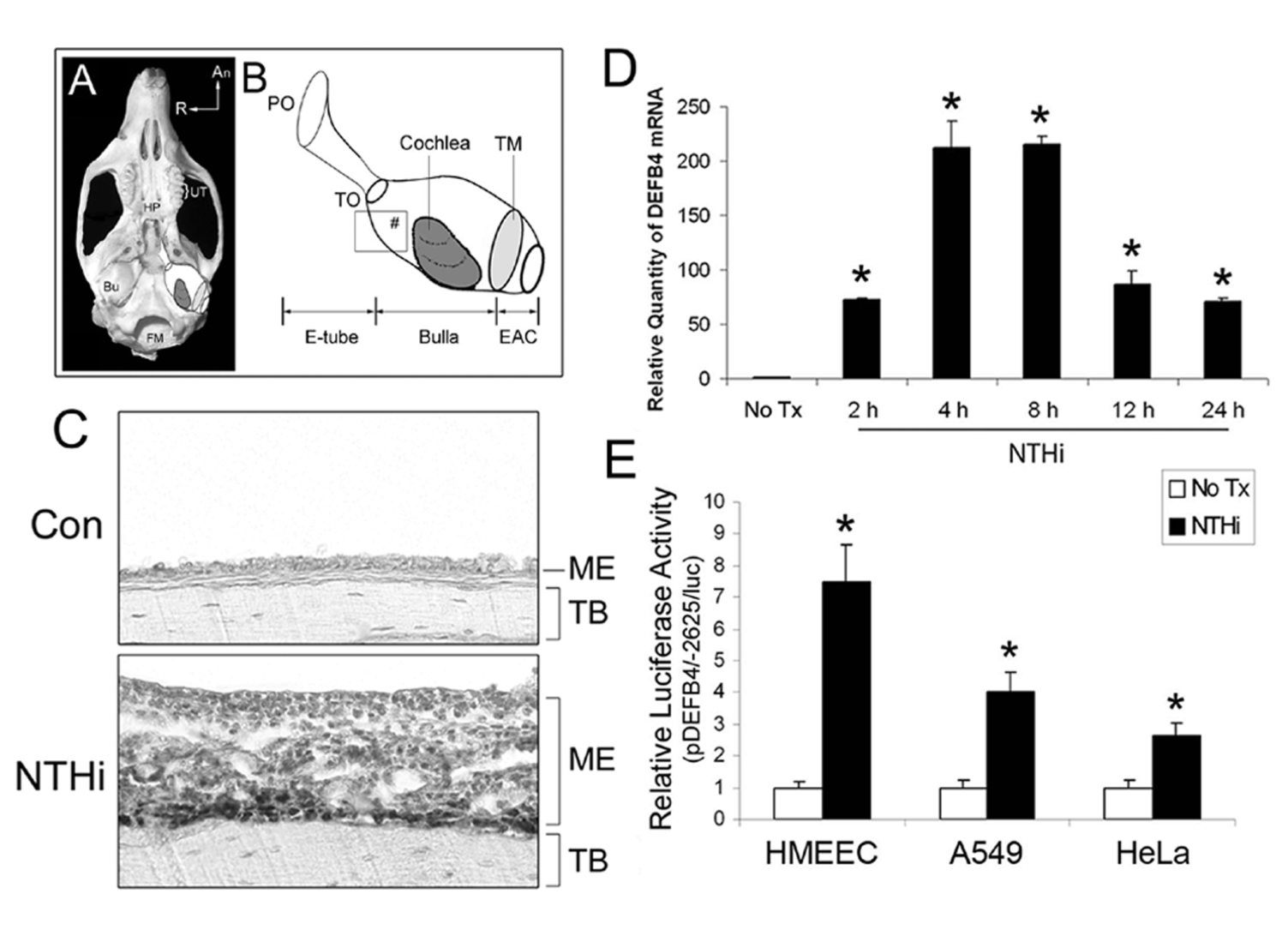


**Figure S1. NTHi induces β-Defensin 2 up-regulation in the middle ear epithelial cells.** (A) An inferior view of the mouse skull base shows that the anatomic relationship of the bulla and Eustachian tube. HP: hard palate, UT: upper teeth, Bu: bulla, FM: foramen magnum, An: anterior, R: right. (B) Schematic illustration of tubotympanum. PO: pharyngeal orifice of Eustachian tube, TO: tympanic orifice of Eustachian tube, TM: tympanic membrane, EAC: external auditory canal, #: the mucosal area nears the tubal orifice for immunolabeling. (C) Immunolabeling shows increased production of mouse Defb2 upon exposure to live NTHi with infiltration of inflammatory cells and mucosal edema. Con: a saline-treated control, ME: middle ear mucosa, TB: temporal bone. Original magnification: x200. (D) Quantitative RT-PCR analysis shows that the NTHi lysate up-regulates human β-defensin 2 in a time dependent manner in the HMEEC cells. E) Luciferase assays show that the NTHi lysate up-regulates human β-defensin 2 expression in the human epithelial cell lines such as the HMEEC cells, the A549 cells and the HeLa cells. DEFB4: human β-defensin 2, Tx: treatment. Results were expressed as fold-induction, taking the value of the non-treated group as 1. Values are given as the mean ± standard deviation (n=3). *: *p*<0.05.
