# Supplementary material for: NOD2/RICK-Dependent β-Defensin 2 Regulation Is Protective for Nontypeable Haemophilus influenzae-Induced Middle Ear Infection
Source: PLoS One. 2014 Mar 13;9(3):e90933. doi: 10.1371/journal.pone.0090933 (PMC3953203; doi:10.1371/journal.pone.0090933)
Supplement: Figure S2 — siRNA-mediated silencing of NOD1, NOD2 and TLR2. Quantitative RT-PCR analysis (A, B) and immunoprecipitation (C) are showing siRNA-mediated silencing of NOD1, NOD2 and TLR2 in the HMEEC cells. NC: a control group silenced with a nonspecific negative control siRNA, KD: a group silenced with a gene-specific siRNA, Double KD: simultaneous silencing of NOD2 and TLR2, IP: immunoprecipitation, WB: western blotting. (DOCX) [file pone.0090933.s002.docx]

**Figure S2. siRNA-mediated silencing of NOD1, NOD2 and TLR2.** Quantitative RT-PCR analysis (A, B) and immunoprecipitation (C) are showing siRNA-mediated silencing of NOD1, NOD2 and TLR2 in the HMEEC cells. NC: a control group silenced with a nonspecific negative control siRNA, KD: a group silenced with a gene-specific siRNA, Double KD: simultaneous silencing of NOD2 and TLR2, IP: immunoprecipitation, WB: western blotting.
