# Supplementary material for: NOD2/RICK-Dependent β-Defensin 2 Regulation Is Protective for Nontypeable Haemophilus influenzae-Induced Middle Ear Infection
Source: PLoS One. 2014 Mar 13;9(3):e90933. doi: 10.1371/journal.pone.0090933 (PMC3953203; doi:10.1371/journal.pone.0090933)
Supplement: Figure S3 — TLR2 and NOD2 are required for NTHi-induced human β-defensin 2 up-regulation. (A) RT-PCR analysis shows that TLR2 deficiency (TLR2−/−) does not completely block NTHi lysate-induced mouse Defb2 up-regulation in the mouse middle ear epithelial cells. WT: wild type, 18s: 18s rRNA. (B) NTHi lysate-induced human β-defensin 2 up-regulation is blocked when both TLR2 and NOD2 are simultaneously silenced in the HMEEC cells. NC: a control group silenced with a nonspecific negative control siRNA, KD: a group silenced with a gene-specific siRNA. (DOCX) [file pone.0090933.s003.docx]

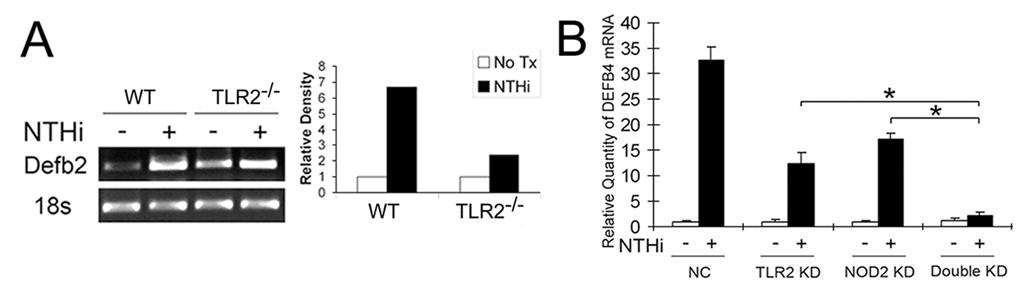


**Figure S3. TLR2 and NOD2 are required for NTHi-induced human β-defensin 2 up-regulation.** (A) RT-PCR analysis shows that TLR2 deficiency (TLR2^-/-^) does not completely block NTHi lysate-induced mouse Defb2 up-regulation in the mouse middle ear epithelial cells. WT: wild type, 18s: 18s rRNA. (B) NTHi lysate-induced human β-defensin 2 up-regulation is blocked when both TLR2 and NOD2 are simultaneously silenced in the HMEEC cells. NC: a control group silenced with a nonspecific negative control siRNA, KD: a group silenced with a gene-specific siRNA.
