# Supplementary material for: NOD2/RICK-Dependent β-Defensin 2 Regulation Is Protective for Nontypeable Haemophilus influenzae-Induced Middle Ear Infection
Source: PLoS One. 2014 Mar 13;9(3):e90933. doi: 10.1371/journal.pone.0090933 (PMC3953203; doi:10.1371/journal.pone.0090933)
Supplement: Figure S4 — (A) RT-PCR analysis shows that mouse Defb4 is up-regulated in response to the NTHi lysate in the primary middle ear epithelial cells derived from wild type mice (WT). In contrast, it is noted that NOD2 deficiency (NOD2−/−) inhibits NTHi-induced up-regulation of Defb4. 18s: 18s rRNA. (B) Quantitative RT-PCR analysis shows that the HMEEC cells up-regulate human β-defensin 2 expression in response to live NTHi in a dose-dependent manner, which fails to show a statistical significance due to a high variance. (C) Note that human β-defensin 2 is up-regulated by the lysate (5.75±1.25 µg/ml) extracted from 5×10^7^ CFU of NTHi more significantly than the same CFUs of live NTHi. *: p<0.05. MTT assays (D) and MultiTox-Glo™ Multiplex Cytotoxicity Assays (Promega) (E) show that the viability of the HMEEC cells is not affected by exposure of 500 ng/ml of α-hemolysin for 5 h. *: p<0.05. (DOCX) [file pone.0090933.s004.docx]

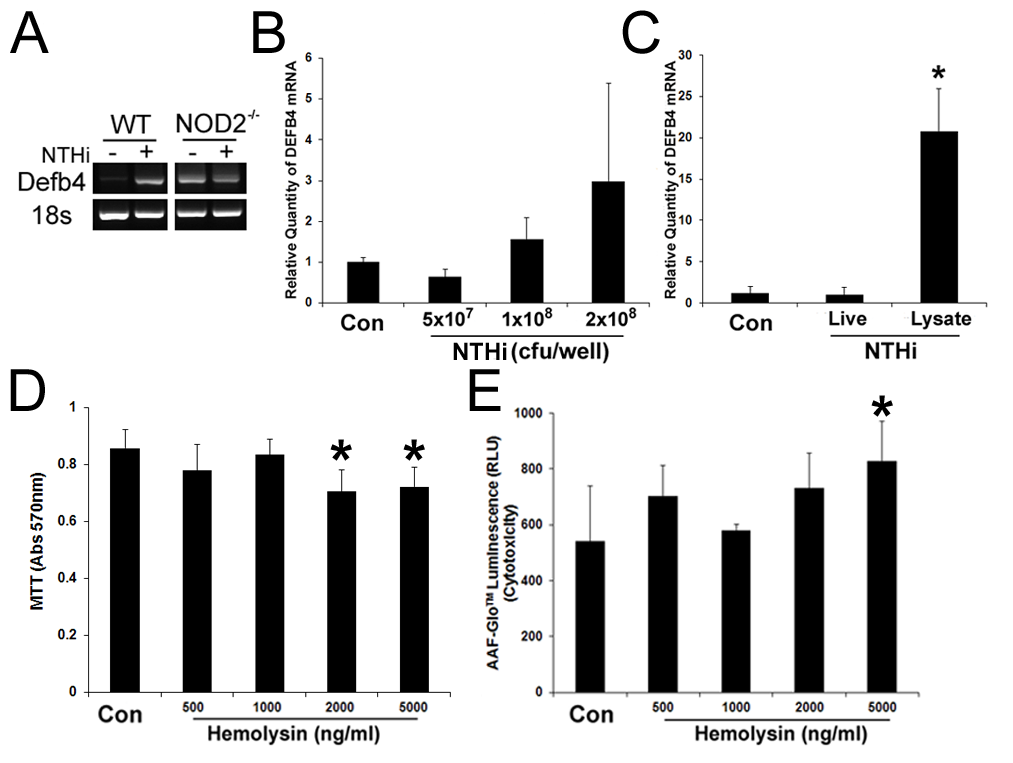


**Figure S4.** (A) RT-PCR analysis shows that mouse Defb4 is up-regulated in response to the NTHi lysate in the primary middle ear epithelial cells derived from wild type mice (WT). In contrast, it is noted that NOD2 deficiency (NOD2^-/-^) inhibits NTHi-induced up-regulation of Defb4. 18s: 18s rRNA. (B) Quantitative RT-PCR analysis shows that the HMEEC cells up-regulate human β-defensin 2 expression in response to live NTHi in a dose-dependent manner, which fails to show a statistical significance due to a high variance. (C) Note that human β-defensin 2 is up-regulated by the lysate (5.75±1.25 μg/ml) extracted from 5x10^7^ CFU of NTHi more significantly than the same CFUs of live NTHi. *: *p*<0.05. MTT assays (D) and MultiTox-Glo^TM^ Multiplex Cytotoxicity Assays (Promega) (E) show that the viability of the HMEEC cells is not affected by exposure of 500 ng/ml of α-hemolysin for 5 h. *: *p*<0.05.
